# Cross-cultural adaptation and psychometric evaluation of a German version of the Activity Patterns Scale (APS-GE) in a large sample of patients with chronic musculoskeletal pain

**DOI:** 10.3389/fpain.2025.1570432

**Published:** 2025-06-13

**Authors:** Anne Kästner, Margarete Donhauser, Inga von Freytag-Löringhoff, Frank Petzke

**Affiliations:** Department of Anesthesiology, Pain Clinic, University Hospital, Georg-August-University of Göttingen, Göttingen, Germany

**Keywords:** test-retest reliability, internal consistency, factor structure, criterion validity, construct validity, avoidance, pacing, persistence

## Abstract

**Background:**

Acknowledging the multidimensionality of pain-related activity patterns led to the development of a new self-report instrument, the Activity Patterns Scale (APS), linking activity pacing to underlying goals. Owing to the scarcity of validated instruments assessing different dimensions of pain-related avoidance, persistence, and pacing behaviors in Germany, our aim was to develop a German version, the APS-GE and to evaluate its psychometric properties in a representative sample of patients with chronic musculoskeletal pain.

**Methods:**

The APS was translated and culturally adapted following the multistep approach recommended by the American Association of Orthopedic Surgeons Outcomes Committee. A comprehensive psychometric evaluation was carried out in 579 patients suffering from chronic musculoskeletal pain. To assess test-retest reliability, the APS-GE was administered twice to a subgroup of patients. Structural validity was tested using covariance and confirmatory factor analysis. To investigate construct and criterion validity, hypotheses were formulated based on the existing literature addressing expected correlations between APS-GE subscales and established questionnaires, and correlations between activity patterns and several functional and psychological outcomes.

**Results:**

Activity patterns varied regarding their test-retest stability. Factor analysis confirmed the multidimensional 8-factor structure proposed previously. For most APS-GE subscales, acceptable construct validity was demonstrated. Interestingly, only 62.5% of hypotheses describing expected associations of activity patterns with functional and psychological outcomes (criterion-related validity) could be confirmed.

**Conclusions:**

The APS-GE appears to be a change-sensitive instrument for the multidimensional assessment of pain-related activity patterns. Remaining conceptual ambiguities should be reevaluated in future studies. Discrepancies to previous investigations regarding the adaptivity of activity patterns could be due to methodological variations across studies. Preliminary implications for putative motivational mechanisms underlying behavioral dimensions are discussed.

## 1 Introduction

How people respond to increases in pain while going about daily activities has a significant impact on their quality of life (1). It is an essential task of interdisciplinary, multimodal pain therapy (IMPT) to change pain-related activity patterns that negatively impact clinical outcomes (2). Pain-related activity patterns are conceptualized as consistent ways of dealing with daily demands in the face of persistent pain (3). As outlined by the Avoidance-Endurance Model (1), these patterns play a pivotal role in the development and maintenance chronic, musculoskeletal pain. Traditionally, three activity patterns have been differentiated in individuals with chronic pain: avoidance, persistence, and pacing. The avoidance pattern involves refraining from activities associated with increases in pain. Persistence has been defined as continuing with activities despite pain. Pacing implies several strategies aimed at creating a balance between the completion of tasks and the avoidance of physical (and mental) overload: taking regular (and pain-independent) recovery breaks or a general reduction of workload or tempo. The valid assessment of pain-related activity patterns in individuals with chronic musculoskeletal pain is of high clinical relevance as state-of-the-art pain management programs (IMPT) which aim to replace dysfunctional coping strategies by more adaptive ones. Self-report instruments useful for the monitoring of therapeutic progress should be sensitive to change and differentiate behaviors conductive to daily functioning from maladaptive ones. It appears well established that the avoidance of physical activities due to anticipated increases in pain is associated with negative functional and psychosocial outcomes (4). A more complex picture has been revealed for persistence and pacing behavior, however. For these activity patterns, substantial variation in the relationships to positive and negative outcomes depending on their operationalization has been demonstrated (4, 5). While excessive overactivity (e.g., doing too much and feeling exhausted afterwards) has been associated with poorer outcomes, items assessing task-contingent persistence conveyed opposite results: completing certain activities irrespective of current pain levels seems to be linked to positive affect and better functioning (5, 6). Interestingly, items across existing questionnaires of activity pacing link different pacing strategies to various underlying purposes (e.g., energy conservation vs. pain reduction). This likely contributes to inconclusive or contradictory results regarding their adaptivity (7).

Recognizing the multidimensionality of avoidance, persistence, and pacing informed the development of the Activity Patterns Scale (APS) (5). Besides differentiating separate dimensions of avoidance and persistence relying on operant factors (task- vs. pain-contingency), a comprehensive content analysis by Nielson et al. (7) postulated three pacing subscales linking pacing strategies to distinct underlying goals (i.e., pacing with the aim of getting more things done, pacing with the aim of reducing pain, pacing with the aim of conserving energy for valued activities). Preliminary findings using the APS indicate that the intentions motivating pacing behavior seem to play an important role with respect to their adaptivity (5).

This psychometric study forms part of a larger research project investigating motivational processes underlying pain-related activity patterns. In the absence of a German questionnaire assessing excessive overactivity and different dimensions of activity pacing in 2021, we translated the APS into German language and pretested the German version (APS-GE) for comprehensibility (study phase 1). Then, we reviewed its psychometric properties in individuals with chronic primary or secondary musculoskeletal pain of different etiologies with special emphasis on criterion validity to get hints on the adaptivity or maladaptivity of certain behavioral dimensions (study phase 2). For the evaluation of construct and criterion validity, a hypothesis testing approach was adopted. In addition, based on previous evaluations (5, 8), we hypothesized that:

a. Test-retest reliabilities for the APS-GE subscales over a time interval of two weeks would be moderate due to fluctuating contextual influences (i.e., pain intensity, motivational factors).
b. The six- and eight factor solutions proposed by Esteve et al. (5) would achieve the best model fit.
c. Internal consistency of APS-GE subscales would vary between 0.60 to 0.80, and corrected item-factor correlations would exceed 0.40.

To explore the relative contributions of APS-GE activity patterns and comparable constructs derived from existing instruments to the explanation of variance in pain-relevant outcome domains (functional impairment and psychological distress), two hierarchical multiple regression analyses were performed.

## 2 Methods

### 2.1 Translation, cultural adaptation, and pretest of the APS-GE

This cross-sectional psychometric study consisted of two phases. In study phase 1, the original version of the APS underwent forward (Spanish to German) and backward translation (German to Spanish) as well as cross-cultural adaptation by an expert translation committee following the multistep approach recommended by the American Association of Orthopedic Surgeons Outcomes Committee (9). In September 2020, we obtained permission from the first author of the original instrument for this project. To evaluate comprehensibility and applicability of the APS-GE, a pretest was performed with 15 volunteers diagnosed with chronic musculoskeletal pain who were recruited from the Pain Clinic of the University Medical Center Göttingen. It was approved by the Ethics Committee of the University Medical Center Göttingen (35/12/21). The pretest was carried out following an adapted procedure of the Three-Step Test-Interview (10). Based on the results of the pretest, the following alterations were made: instructions were simplified, and four statements, as well as frequency terms underwent linguistic adjustments to adapt them to everyday German language. The changes were discussed consensually within the expert translation committee leading to the final version of the APS-GE. A detailed description of the steps involved in the translation and cultural adaptation is given in Supplementary Material S1. The German version of the APS (APS-GE) is presented in Supplementary Material S2.

### 2.2 Psychometric evaluation of the APS-GE

The psychometric evaluation of the APS-GE was carried out according to the COSMIN recommendations (11), the declaration of Helsinki, and the validation of the original version of the instrument by Esteve et al. (5). The psychometric study was registered at the German Clinical Trials Register (DRKS-ID DRKS00035996). It forms part of a larger research project on the motivational underpinnings of pain-related activity patterns based at the Pain Clinic of the University Medical Center Göttingen and was approved by the Ethics Committee of the University Medical Center Göttingen (30/8/21), and the Ärztekammer Niedersachsen (Ar/189/2021).

#### 2.2.1 Participants and recruitment

Participants for this cross-sectional cohort study were recruited from October 2021 to October 2023 at the Pain Clinic of the University Medical Center Göttingen and collaborating registered practitioners specialized in pain medicine and rheumatology. Consecutive patients seeking routine care (i.e., pain and/or rheumatological management) were screened for eligibility by their respective physician. Eligible participants were those aged 18 to 80 years of any gender with primary and/or secondary musculoskeletal pain (including tension-type headaches) according to ICD-11 (12) who had been experiencing persistent or recurrent pain for at least six months. They had to be willing to take part in the study, having sufficient German literacy skills to extract meaning from written materials, and be cognitively and emotionally resilient enough to complete the questionnaire package. Participants with rheumatoid arthritis should be on stable medication regimen and in disease remission at the time of their consultation. Patients with a prevailing neuropathic pain component (e.g., polyneuropathy, nerve injury), or severe psychiatric or neurological comorbidities (e.g., addiction disorder, acute psychotic symptoms, severe depressive episode, dementia) were excluded. Eligible patients were informed about the study by their physician and asked to contact the study team if they were interested in participating. Sample size was determined *a priori* based on the recommendations for factor analysis (FA), suggesting 4 to 10 participants per item and more than 100 data sets in total (13).

#### 2.2.2 Clinical subgrouping of patients with chronic musculoskeletal pain

Participants were classified into clinical subgroups based on the main pain-related diagnosis and clinical reasoning involving ICD-11 diagnostic criteria (12), etiological considerations, and patient-reported information about e.g., the anatomical distribution and pain characteristics extracted from the German pain questionnaire (14, 15). The German pain questionnaire is a multidimensional instrument for the assessment of chronic pain comprising validated instruments such as the Veterans RAND 12-Item Health Survey (VR-12) (16). The following six subgroups were defined which we hypothesized to differ with respect to the extent of a modulation of the subjective pain experience by central processing and behavioral factors:

a. Upper and lower extremity pain: Patients with localized pain in one well-defined part of an extremity
b. Non-specific neck and/or back pain: Participants with chronic musculoskeletal pain in the back and/or neck region
c. Multisite pain: Patients suffering from chronic pain at a minimum of three different body sites with diverse underlying somatic etiologies (e.g., impingement syndrome of the shoulder, knee osteoarthritis, and bursitis)
d. Rheumatoid arthritis (RA) and other inflammatory diseases: Patients with rheumatoid arthritis and related diagnoses in remission as determined by the responsible physician specialized in rheumatology
e. Fibromyalgia syndrome (FMS): Patients with fibromyalgia as the primary diagnosis. Fibromyalgia was determined by the preliminary 2011 ACR criteria, including a full clinical assessment (17, 18)
f. RA and FMS: Patients with RA in remission and comorbid “secondary” FMS (19)

#### 2.2.3 Study procedure

Patients considered eligible were provided with initial information about the study during their outpatient appointments at the recruitment facilities and were asked about their willingness to participate. Those interested in participating contacted the study team (MD, AK, IFL, FP) and were provided with detailed information about the study procedure and data protection issues. All remaining questions were carefully addressed by the study team. After giving their informed consent, a brief medical history interview was conducted to collect essential sociodemographic and clinical data, including age, gender identity, marital status, education, current employment status, pain-related diagnoses, primary pain diagnoses, duration of musculoskeletal pain, current pain medication, and any prior pain or psychotherapeutic treatments.

Subsequently, a questionnaire package containing the APS-GE, the Fibromyalgia Survey Questionnaire *(FSQ)* and instruments relevant for the evaluation of construct and criterion validity (see below) was handed out. Participants were free to decide whether to complete the questionnaire package on site or at home and return it in a postage-prepaid envelope. Upon returning the questionnaire package and confirming its completeness, patients received compensation of 20 Euros. In case of mistakes or omissions, participants were contacted via telephone to provide the missing information. The collected data was transferred to SPSS for statistical analyses.

#### 2.2.4 Test-retest reliability

To evaluate test-retest reliability of APS-GE subscales, a subgroup of participants was asked to complete the APS-GE for a second time two weeks after filling out the first copy of the questionnaire. Although we expected some of the behaviors to be context-sensitive and thus likely to change within short periods of time (hours or days), a time interval of two weeks was chosen to exclude memory effects. A sample size of 55 individuals was determined adequate, expecting ICCs (intraclass-correlation-coefficient) of 0.6 to 0.8 with a significance level α of 0.05 (two-tailed), 80% power and a 10% drop-out rate (20).

#### 2.2.5 Instruments

##### 2.2.5.1 APS

The APS (5) is a self-report questionnaire constructed by factor analysis from existing instruments assessing avoidance and endurance behaviors and theoretical considerations (5). Three activity pacing subscales were developed for the APS based on a construct analysis by Nielsen et al. (21) and subjected to a pretest to exclude comprehension problems. In summary, the APS consists of 24 items assigned to 8 three-item subscales: pain avoidance, activity avoidance, task-contingent persistence, excessive overactivity, pain-contingent persistence, pacing aimed at increasing activity levels, pacing aimed at conserving energy for valued activities, and pacing aimed at reducing pain. The frequency of performing the activities is rated on a five-point Likert scale from 0 = “never” to 4 = “always”. High scores correspond to high levels of the respective behavioral dimension.

##### 2.2.5.2 Fibromyalgia survey questionnaire (FSQ)

The FSQ is a self-administered instrument to classify FMS in survey research without a physical examination. It comprises two subscales: the Widespread Pain Index (WPI), which assesses pain or tenderness at 19 different body parts, resulting in a total score between 0 and 19 and the Somatic Severity Score (SSS). The SSS captures the somatic symptom burden by inquiring about fatigue, trouble thinking, tiredness after waking up, pain in the lower abdomen, depression, and headache. SSS total scores range from 0 to 12. We used the WPI in this study to validate the clinical subgrouping (i.e., subgroups “multisite pain”, “RA” and “FMS” should have higher WPI scores than the subgroups “upper and lower extremity pain” and “non-specific back and/ or neck pain”) and to approximate the somatic symptom load. The German version has been validated by Häuser et al. (22).

##### 2.2.5.3 Instruments for the evaluation of construct validity

###### 2.2.5.3.1 Pain Catastrophizing Scale (PCS)

The PCS (23) assesses the construct of catastrophizing with conceptual proximity to fear-avoidance behavior (24). It contains 13 statements describing rumination and magnification of worries as well as feelings of helplessness (i.e., catastrophizing). Items are rated on a 5-point Likert-type scale ranging from 0 = “does not apply at all” to 4 = “always applies”. The sum score ranging from 0 to 52 reflects a person's overall catastrophizing tendency was used in this study. The German version of this instrument has shown comparable psychometric properties to the English version (25).

###### 2.2.5.3.2 Fear-Avoidance-Beliefs Questionnaire (FABQ)

The FABQ is a self-report questionnaire designed to assess pain and activity avoidance in patients (26). The FABQ was originally developed for patients suffering from chronic low back pain. We adapted the instruction such that patients were asked to refer to other pain sites as well. It comprises 16 statements addressing the impact of physical activities, such as bending, lifting, or walking, and occupational activities on the pain experience. The degree of accuracy of these statements is to be evaluated on a seven-point Likert scale ranging from 0 = “not at all true” to 6 = “completely true”. A higher total sum score indicates a stronger belief in one's own pain symptoms being caused or exacerbated by physical activities and/or one's own occupational activities. The subscale assessing “activity avoidance” was used in this study. The German version of the questionnaire demonstrates robust psychometric properties (27).

###### 2.2.5.3.3 Avoidance-Endurance-Questionnaire (AEQ)

The AEQ (28) assesses cognitive, emotional, and behavioral fear-avoidance and endurance responses to mild and severe chronic low back pain. It is grounded in the Avoidance-Endurance Model proposed by Hasenbring et al. (1). The AEQ comprises three subscales: The AEQ-ERSS (Emotional Reactions to Strong Pain) subscale is made up of 10 adjectives describing the emotional state of the past 14 days in response to severe pain symptoms. The AEQ-KRSS (Cognitive Reactions to Strong Pain) section comprises 16 statements describing cognitive responses to episodes of severe pain. The third subscale, AEQ-CRSS (Coping Reactions to Strong Pain), consists of 23 items describing different pain coping behaviors. All statements are to be rated on a 7-point Likert-scale ranging from 0 = “never” to 6 = “always”. For the evaluation of construct validity, the sum scores “avoidance of physical activities” (5 items), “avoidance of social activities” (6 items), and “endurance” (11 items) of the AEQ-CRSS subscale were used. Higher values reflect more frequent behaviors in coping with episodes of severe pain. The questionnaire demonstrates robust psychometric qualities (28).

##### 2.2.5.4 Instruments for the evaluation of criterion validity

###### 2.2.5.4.1 Chronic Pain Grade Questionnaire (CPGQ)

The CPGQ (29) assesses the subjective severity of chronic pain (intensity and pain-related impairment) over the past three months. First, the intensity of their current, average, and strongest pain is to be rated on a Numerical Rating Scale (NRS) from 0 = “no pain” to 10 = “worst imaginable pain” each. The average of the three pain intensity ratings was used for the analysis of criterion validity. Then, patients are asked to indicate the number of days they felt incapable of going to work because of their pain in the past three months (“days of incapacity for work”). Additionally, the pain-related functional impairment over the past three months in three different functional domains (everyday activities, leisure activities, and wort-related activities) is measured on an NRS from 0 = “no impairment” to 10 = “maximum impairment”. The average of these three ratings was used for the analyses. Good psychometric qualities are reported for the German version (30).

###### 2.2.5.4.2 Pain Disability Index (PDI)

The PDI (31) is a self-report questionnaire assessing the subjective extent of impairment on a Numerical Rating Scale (NRS from 0 = “no impairment” to 10 = “maximum impairment”) across seven life domains: 1. family and household responsibilities, 2. recreation, 3. social activities, 4. occupation, 5. sexual life, 6. self-care, and 7. essential activities. The higher the sum score of all seven items, the greater the perceived pain-related impairment. Satisfactory psychometric qualities of the German version have been documented (32).

###### 2.2.5.4.3 Depression Anxiety Stress Scale (DASS-21)

The DASS-21 (33) is a 21-item self-report questionnaire measuring psychological distress in terms of typical symptoms of depression, anxiety and stress. It consists of three subscales (“depression”, “anxiety”, and “stress”), each comprising seven statements to be rated on a 4-point Likert-type scale (0 = “did not apply at all” to 3 = “applied very much”) with higher values indicating higher psychological distress. The German version of the DASS-21 has acceptable psychometric properties (34).

###### 2.2.5.4.4 Positive and Negative Affect Schedule (PANAS)

The PANAS Scale (35) is a self-report questionnaire comprising two subscales designed for the global recording of positive and negative affective states. The “Positive Affect (PA)” subscale denotes an enthusiastic, active, and alert state, whereas the “Negative Affect (NA)” dimension describes a state of negative tension through feelings of sadness, anger, and fear. Both subscales consist of ten adjectives each representing mood states, such as “irritable” (NA subscale) or “determined” (PA subscale), whose present intensity is rated on a five-point Likert-type scale ranging from 1 = “not at all” to 5 = “extremely”. High values correspond to elevated levels of the respective affective dimension. The German version of the PANAS scale demonstrates satisfactory psychometric qualities comparable to the original version (36).

###### 2.2.5.4.5 Veterans Rand 12-Item Health Survey (VR-12)

The VR-12 (37) was constructed to measure two essential dimensions of health-related quality of life (QoL) by 12 statements related to seven health domains: general health, physical functioning, physical role functioning, mental health, mental role functioning, limitations due to pain, and social functioning. Two composite scores can be derived based on weighted linear combinations of the total set of items, which are transformed into T scores (mean 50, standard deviation 10). The Physical Component Summary Score (PCS) is indicative of general health perception, physical role functioning, and pain while the Mental Component Summary Score (MCS) is supposed to reflect emotional role functioning, mental well-being, negative affectivity, and social functioning. Higher PCS or MCS values correspond to a higher health-related physical or mental QoL. A validated German version of the VR-12 (16) is included in the German pain questionnaire (15).

#### 2.2.6 Statistical analyses

Data analyses were performed with IBM SPSS Statistics for Windows, version 29.0 (Armonk, NY, USA: IBM Corp.) and R software version 4.2.2, lavaan package 0.6–12 (https://www.r-project.org/).

For all statistical analyses, significance level was set to α = 0.05. To account for multiple testing, Bonferroni corrections of significance levels were applied as indicated in Table and Figure legends. Correlation coefficients were interpreted according to Cohen (38).

We described continuous variables by mean and standard deviation, ordinal variables by median and interquartile range. Discrete variables were presented as absolute numbers and frequencies. The distribution of continuous data was tested for normality using the Kolmogorov–Smirnov test. We evaluated differences between clinical subgroups by Kruskal–Wallis tests and Dunn–Bonferroni *post-hoc* comparisons (ordinal variables or in case of violations of prerequisites for parametric approaches), or Chi-square tests (discrete variables). To be able to attribute statistically significant summary statistics from chi-square tests to differences between actual and expected frequencies of single variable categories and clinical subgroups, adjusted standardized residuals from cross tabulations were converted to *p*-values using the chi-square distribution (Tables 1–3).

**Table 1:** Sociodemographic description of the complete study sample and clinical subgroups.

	Complete study sample (*N* = 579)	Upper or lower extremity pain (*N* = 56)	Non-specific neck and back pain (*N* = 240)	Multisite pain (*N* = 82)	RA & other inflamm. cond. (*N* = 68)	FMS (*N* = 114)	RA & FMS (*N* = 17)	Test statistic (*p*-value^a^)
Age in years (mean ± SD)	53.8 ± 12.2	51.9 ± 12.8	54.7 ± 12.2	50.7 ± 14.3	54.3 ± 11.8	54.5 ± 10.6	54.5 ± 9.9	H = 6.67 (*p* = .154)
Gender identity, *N* (%)								
Female	433 (74.8)	34 (60.7)	170 (70.8)	60 (73.2)	48 (70.6)	103 (90.4)	16 (94.1)	*χ*² = 26.46 (***p* < .001**)^b^^,^^c^
Male	146 (25.2)	22 (39.3)	20 (29.4)	22 (26.8)	20 (29.4)	11 (9.6)	1 (5.9)	
Transgender, non-binary	0	0	0	0	0	0	0	
Highest school-leaving qualification, *N* (%)								
None	4 (.7)	0	2 (.8)	0	1 (1.5)	1 (.9)	0	χ² = 21.01 (*p* = .178)
Low^d^	129 (22.3)	8 (14.3)	60 (25.0)	20 (24.4)	16 (23.5)	21 (18.4)	4 (23.5)	
Intermediate^e^	257 (44.4)	24 (42.9)	107 (44.6)	24 (29.3)	32 (47.1)	63 (55.3)	5 (29.4)	
Technical high school^f^	55 (9.5)	7 (12.5)	17 (7.1)	13 (15.9)	6 (8.8)	9 (7.9)	3 (17.6)	
General high school^g^	134 (23.1)	17 (30.4)	54 (22.5)	25 (30.5)	13 (19.1)	20 (17.5)	5 (29.4)	
Marital status, *N* (%)								
Single	109 (18.8)	13 (23.2)	37 (15.4)	19 (23.2)	16 (23.5)	22 (19.3)	1 (5.9)	χ² = 18.48 (*p* = .102)
In a relationship/married	425 (73.4)	43 (76.8)	176 (73.3)	54 (65.9)	51 (75.0)	84 (73.7)	16 (94.1)	
Separated/divorced	29 (5.0)	0	17 (7.1)	6 (7.3)	0	6 (5.3)	0	
Widowed	16 (2.8)	0	10 (4.2)	3 (3.7)	1 (1.5)	2 (1.8)	0	
Current employment status, *N* (%)								
Full-time employment	151 (26.1)	20 (35.7)	65 (27.)	21 (25.6)	22 (32.8)	21 (18.4)	2 (11.8)	χ² = 36.97 *(p* *=* *.012)*
Part-time employment	161 (27.9)	7 (12.5)	67 (27.9)	31 (37.8)	20 (29.9)	29 (25.4)	5 (29.4)	
Unemployed	27 (4.7)	6 (10.7)	13 (5.4)	2 (2.4)	2 (3.0)	3 (2.6)	1 (5.9)	
Incapacitated for work	44 (7.6)	8 (14.3)	19 (7.9)	6 (7.3)	3 (4.5)	7 (6.1)	1 (5.9)	
Applying for pension	16 (2.7)	1 (1.8)	5 (2.1)	3 (3.7)	2 (2.9)	4 (3.5)	1 (5.9)	
Retired	180 (31.0)	14 (25.0)	71 (29.6)	19 (23.2)	19 (28.4)	50 (43.9)	7 (41.2)	

RA, rheumatoid arthritis; FMS, fibromyalgia.

^a^ Significance level after Bonferroni correction:.05/5 = .01, statistically significant *p*-values in bold, nominally significant *p*-values in italics.

^b^ To be able to attribute statistically significant summary statistics from chi-square test to differences between actual and expected frequencies of single variable categories and diagnostic subgroups, adjusted standardized residuals from cross tabulations were converted to *p*-values using the chi-square distribution (significance level of *p* = .05 was divided by the number of cells to adjust for multiple testing).

^c^ Less male FMS patients than expected.

^d^ Low secondary school-leaving certificate (“Hauptschulabschluss”, qualifies for vocational training).

^e^ Intermediate secondary school-leaving certificate (“Realschulabschluss”, qualifies for vocational training, technical and general high-school, and universities of applied sciences).

^f^ Technical high-school diploma (“Fachabitur”, qualifies for specific university study programs).

^g^ General high-school diploma (“Abitur”, qualifies for any university study program).

**Table 2:** Clinical description of the complete study sample and clinical subgroups.

	Complete study sample (*N* = 577–579)^a^	Upper or lower extremity pain (*N* = 55–56; A)	Non-specific neck and back pain (*N* = 240; B)	Multisite pain (*N* = 82; C)	RA & other inflamm. cond. (*N* = 67–68; D)	FMS (*N* = 114; E)	RA & FMS (*N* = 17; F)	Test statistic (*p*-value^b^)	Sign. pairwise *post-hoc* comparisons^c^^,^^d^
Duration of pain in months (mean ± SD)	146.8 ± 126	82.9 ± 86.4	136.8 ± 131.2	165.9 ± 122.5	141.0 ± 117.2	185.7 ± 129.3	156.7 ± 75.5	H = 39.94 (***p* < .001**)	A vs. all other groups; B vs. E
Widespread pain index (mean ± SD)	7.2 ± 4.1	4.9 ± 3.3	6.2 ± 3.5	7.3 ± 4.1	6.2 ± 2.5	10.5 ± 3.6	13.1 ± 4.7	H = 130.00 (***p* < .001**)	C vs. A; E vs. all other groups
Somatic symptom severity (mean ± SD)	6.8 ± 2.7	6.5 ± 3.1	6.3 ± 2.7	6.7 ± 2.7	6.3 ± 2.5	8.2 ± 2.3	7.6 ± 2.9	H = 42.5 (***p* < .001**)	E vs. all other groups
Drug regimen, *N* (%) continuous medication	332 (57.5)	30 (53.6)	127 (52.9)	35 (42.7)	53 (77.9)	72 (63.2)	14 (82.4)	χ² = 25.39 (***p* < .001**)	C: Less cases on continuous meds than expected; D: More cases on continuous meds than expected
Pain relief by analgesics, *N* (%) pain relief	430 (74.5)	38 (69.1)	171 (71.3)	64 (79.0)	58 (85.3)	85 (74.6)	12 (70.7)	χ² = 17.72 *(p* *=* *.023)*	
Psychiatric pre-treatments, *N* (%)									
None	279 (48.4)	28 (50.0)	127 (53.1)	38 (46.3)	43 (63.2)	36 (31.6)	5 (29.4)	χ² = 36.29 (***p* = .003**)	E: Less cases without prior psychiatric treatment than expected
Inpatient	79 (13.7)	11 (19.6)	28 (11.7)	11 (13.4)	4 (5.9)	23 (20.2)	2 (11.8)		
Day-care	11 (1.9)	3 (5.4)	2 (.8)	1 (1.2)	1 (1.5)	4 (3.5)	0		
Outpatient	208 (36.0)	14 (25.0)	82 (34.3)	32 (39.0)	20 (29.4)	50 (43.9)	10 (58.8)		
Outpatient psychotherapy, *N* (%)									
None	256 (44.4)	27 (48.2)	112 (46.7)	35 (42.7)	40 (58.8)	36 (31.6)	4 (23.5)	χ² = 41.27 (***p* < .001**)	B: Less cases with >50 sessions than expected; E: Less cases with no pre-treatment and more cases with >50 sessions than expected
0–25 Sessions	132 (22.9)	8 (14.3)	66 (27.5)	16 (19.5)	15 (22.1)	22 (19.3)	5 (29.4)		
26–50 Sessions	73 (12.7)	7 (12.5)	30 (12.5)	14 (17.1)	5 (7.4)	15 (13.2)	2 (11.8)		
>50 Sessions	118 (20.5)	14 (25.0)	32 (13.3)	17 (20.7)	8 (11.8)	41 (36.0)	6 (35.3)		
Pre-treatments IMPT, *N* (%)									
None	321 (55.6)	34 (60.7)	118 (49.2)	47 (57.3)	51 (75.0)	58 (50.9)	11 (64.7)	χ² = 26.82 *(p* *=* *.008)*	
Inpatient	51 (8.8)	3 (5.4)	20 (8.3)	5 (6.1)	4 (5.9)	17 (14.9)	2 (11.8)		
Day-care	170 (29.5)	17 (30.4)	88 (36.7)	23 (28.0)	9 (13.2)	29 (25.4)	4 (23.5)		
Outpatient	37 (6.4)	2 (3.6)	14 (5.8)	7 (8.5)	4 (5.9)	10 (8.8)	0		

RA, rheumatoid arthritis; FMS, fibromyalgia.

^a^ Sample sizes vary slightly due to missing data.

^b^ Significance level after Bonferroni correction:.05/7 = .007, statistically significant *p*-values in bold, nominally significant *p*-values in italics.

^c^ Only *p*-values withstanding Bonferroni correction for multiple testing are presented (0.05/number of *post-hoc* comparisons).

^d^ To be able to attribute statistically significant summary statistics from chi-square tests to differences between actual and expected frequencies of single variable categories and diagnostic subgroups, adjusted standardized residuals from cross tabulations (e.g., drug regimen x diagnostic subgroups) were converted to *p*-values using the chi-square distribution (significance level of *p* = .05 was divided by the number of cells to adjust for multiple testing).

**Table 3:** Comparison of clinical subgroups with respect to the type of pain medication.

	Complete study sample (*N* = 578)	Upper or lower extremity pain (*N* = 56)	Non-specific neck and back pain (*N* = 240)	Multisite pain (*N* = 82)	RA & other inflamm. cond. (*N* = 69)	FMS (*N* = 114)	RA & FMS (*N* = 17)	χ² (*p*-value^a^)
Type of long-term, oral pain medication, *N* (%)								
None	69 (11.9)	7 (12.5)	28 (11.7)	12 (14.6)	8 (11.6)	11 (9.6)	3 (17.6)	1.704 (.888)
Non-opioids	407 (70.4)	38 (67.9)	170 (70.8)	58 (70.7)	49 (71.0)	81 (71.1)	11 (64.7)	.500 (.992)
Opioids	201 (34.8)	19 (33.9)	99 (41.3)	24 (29.3)	25 (36.2)	28 (24.6)	6 (35.3)	10.860 (.054)
Antidepressants	109 (18.9)	7 (12.5)	32 (13.3)	14 (17.1)	8 (11.6)	43 (37.7)	5 (29.4)	36.557 (**<.001**)^b^^,^^c^
Anticonvulsants	88 (15.4)	7 (12.5)	42 (17.5)	7 (8.5)	11 (15.9)	19 (16.7)	3 (17.6)	4.361 (.499)
Others	38 (6.6)	7 (12.5)	10 (4.2)	5 (6.1)	7 (10.1)	9 (7.9)	0	8.449 (.133)
Analgesic drug combinations per patient, *N* (%)								
Monotherapy	266 (46.0)	26 (46.4)	113 (47.1)	42 (51.2)	30 (43.5)	48 (42.1)	7 (41.2)	15.177 (.766)
Combination of two analgesic drug classes	164 (28.4)	19 (33.9)	62 (25.8)	20 (24.4)	24 (34.8)	35 (30.7)	4 (23.5)	
Combination of three analgesic drug classes	65 (11.2)	2 (3.6)	32 (13.3)	6 (7.3)	5 (7.2)	18 (15.8)	2 (11.8)	
Combination of four analgesic drug classes	14 (2.4)	2 (3.6)	5 (2.1)	2 (2.4)	2 (2.9)	2 (1.8)	1 (5.9)	

RA, rheumatoid arthritis; FMS, fibromyalgia.

^a^ Significance level after Bonferroni correction:.05/2 = .025, statistically significant *p*-values in bold, nominally significant *p*-values in italics.

^b^ To be able to attribute statistically significant summary statistics from chi-square test to differences between actual and expected frequencies of single variable categories and diagnostic subgroups, adjusted standardized residuals from cross tabulations were converted to *p*-values using the chi-square distribution (significance level of *p* = .05 was divided by the number of cells to adjust for multiple testing).

^c^ More FMS patients on antidepressant medication than expected.

##### 2.2.6.1 Reliability

Internal consistency of the APS-GE subscales was assessed using Cronbach's *α* and corrected item-factor correlations (Table 4). By convention, a Cronbach's α of 0.65 to 0.80 is considered acceptable for scales in human dimensions research (39). Following the recommendations of Vaske et al. (39), corrected item-factor correlations should be equal to or above 0.40. Test-retest reliability of the APS-GE subscales was evaluated by intraclass-correlation-coefficients_2,1_ (ICC_2,1_- two-way mixed effects, absolute agreement, single measurement model). Based on recent recommendations (40), ICC estimates < 0.5 were considered poor, 0.5 to 0.75 moderate, 0.75 to 0.9 good and >0.9 excellent. We could not analyze limits of agreement (e.g., Smallest Detectable Change) using Bland Altman Plots because the differences between test and retest scores per individual were not normally distributed as tested by Shapiro–Wilk tests. Spearman's rank correlation coefficients and polychoric correlation coefficients were calculated to describe stability of single items (Table 5).

**Table 4:** Means, SD, corrected item-factor correlations of the items of the APS-GE.

Factors/Items	Mean	SD	Corrected item-factor correlations	Cronbach's *α* of subscales
Factor I: Pain avoidance				
1. I stop what I am doing when my pain starts to get worse.	1.97	.92	.44	.73
11. If I know that some activity may make my pain worse, I don’t do it anymore.	1.91	1.03	.63	
16. I avoid activities that cause pain.	1.95	1.00	.63	
Factor II: Activity avoidance				
6. I have not been able to carry on with my usual level of activity.	2.53	1.10	.55	.71
8. Because of my pain, most days I spend more time resting than doing other activities.	1.63	1.03	.47	
13. I have to put parts of my life on hold.	2.14	1.04	.56	
Factor III: Task-contingent persistence				
2. Kept on doing what I was doing.	2.70	.89	.62	.84
10. I just kept going.	2.33	1.06	.76	
21. Once I start an activity I keep going until it is done.	2.40	1.03	.74	
Factor IV: Excessive persistence				
4. I have tried to do too much, and I felt even worse as a result.	2.25	.96	.58	.71
7. I find myself rushing to get everything done before I crash.	1.94	1.13	.46	
15. I have overdone things, then I needed to rest for some time.	2.10	.97	.57	
Factor V: Pain-contingent persistence				
18. When my pain decreases, I try to be as active as possible.	2.85	.96	.51	.73
20. I do extra on days my pain is less.	2.49	1.05	.51	
22. I make the most of my good pain days by doing more things.	2.72	.93	.64	
Factor VI: Pacing for the purpose of increasing activity level				
3. I usually take several breaks so I can do a lot more things.	2.13	1.04	.58	.75
17. I do things more slowly so I can do a lot more things.	1.84	1.00	.56	
19. I split activities into smaller parts so I can do a lot more things.	1.84	.97	.60	
Factor VII: Pacing for the purpose of conserving energy for valued activities				
5. I usually take several breaks so I can save energy for other things that matter to me.	1.86	1.01	.60	.77
14. I do things more slowly so I can save energy for other things that matter to me.	1.99	1.01	.62	
23. I split activities into smaller parts so I can save energy for other things that matter to me.	1.82	.94	.61	
Factor VIII: Pacing for the purpose of pain reduction				
9. I usually take several breaks so that it hurts less.	2.06	1.03	.47	.73
12. I do things more slowly so that it hurts less.	1.96	1.00	.57	
24. I split activities into smaller parts so that it hurts less.	1.84	1.08	.62	

**Table 5:** Test-retest reliability of the subscales and items of the APS-GE in a sample subset (*N* = 52).

Subscale	ICC_2,1_^a^^,^^b^ (95% CI)	Item	Spearman's rank correlation			Polychoric correlation
			rho	95% CI	*p*-value	r
Pain avoidance	0.71 (0.54, 0.82)	1	0.61	[0.40, 0.76]	**<0**.**0001**	0.70
		11	0.45	[0.21, 0.65]	**0**.**0008**	0.54
		16	0.65	[0.46, 0.79]	**<0**.**0001**	0.67
Activity avoidance	0.70 (0.53, 0.82)	6	0.65	[0.45, 0.78]	**<0**.**0001**	0.69
		8	0.78	[0.65, 0.87]	**<0**.**0001**	0.82
		13	0.58	[0.36, 0.73]	**<0**.**0001**	0.61
Task-contingent persistence	0.50 (0.27, 0.68)	2	0.25	[−0.02, 0.49]	0.072	0.22
		10	0.54	[0.31, 0.71]	**<0**.**0001**	0.62
		21	0.55	[0.33, 0.72]	**<0**.**0001**	0.63
Excessive persistence	0.62 (0.42, 0.76)	4	0.58	[0.37, 0.74]	**<0**.**0001**	0.66
		7	0.40	[0.14, 0.60]	**0**.**0035**	0.55
		15	0.40	[0.14, 0.61]	**0**.**0034**	0.49
Pain-contingent persistence	0.63 (0.44, 0.77)	18	0.37	[0.11, 0.58]	**0**.**0068**	0.48
		20	0.50	[0.26, 0.68]	**0**.**0002**	0.53
		22	0.52	[0.28, 0.69]	**<0**.**0001**	0.58
Pacing- increasing activity	0.71 (0.55, 0.83)	3	0.55	[0.32, 0.71]	**<0**.**0001**	0.63
		17	0.68	[0.50, 0.80]	**<0**.**0001**	0.77
		19	0.52	[0.29, 0.70]	**<0**.**0001**	0.54
Pacing- conserve energy	0.64 (0.45, 0.78)	5	0.32	[0.05, 0.54]	**0**.**022**	0.37
		14	0.55	[0.32, 0.71]	**<0**.**0001**	0.62
		23	0.62	[0.42, 0.77]	**<0**.**0001**	0.79
Pacing- pain reduction	0.68 (0.50, 0.80)	9	0.56	[0.34, 0.72]	**<0**.**0001**	0.62
		12	0.63	[0.43, 0.77]	**<0**.**0001**	0.73
		24	0.58	[0.37, 0.74]	**<0**.**0001**	0.60

ICC, intraclass-correlation-coefficient; CI, confidence interval; *p*-values set in boldface indicate statistical significance (adjustment for multiple testing by Holm-Bonferroni method).

^a^ Two-way mixed effects, absolute agreement, single measurement model.

^b^ Standard Error of Measurement (SEM) and Smallest Detectable Change (SDC) could not be calculated because the difference between the two scores per APS-GE subscale were not normally distributed.

##### 2.2.6.2 Structural validity

As part of an item and scale analysis of the APS-GE, descriptive statistics (mean and standard deviation) were first determined (Table 4). By calculating correlations (Spearman's rank) of the APS-GE items with their respective subscale average, the discriminatory power of the items was determined as an indicator of their representativeness which should exceed 0.3 to be acceptable (41) (Table 4). In addition, we assessed the covariance structure of the APS-GE subscales (6-factor and 8-factor models) by Spearman's rank correlation (Table 6).

**Table 6:** Covariance structure of the APS-GE subscales (6-factor and 8-factor models).

	Pain avoidance	Activity avoidance	Task-contingent persistence	Excessive persistence	Pain-contingent persistence	Pacing-increasing activity	Pacing-conserve energy	Pacing-pain reduction
Pain avoidance	1							
Activity avoidance	.64	1						
Task-contingent persistence	−.51	−.37	1					
Excessive persistence	ns	.43	.29	1				
Pain-contingent persistence	ns	ns	.29	.41	1			
Pacing (6-factor model)	.47	.48	−.44	−.14	ns	1	1	1
Pacing- increasing activity	.44	.39	−.44	−.19	ns	1		
Pacing- conserve energy	.38	.38	−.39	−.16	.16	1.11^a^	1	
Pacing- pain reduction	.63	.68	−.53	ns	ns	1.07^a^	1.02^a^	1

Only significant correlations (Spearman's rank) after correction for multiple testing (6-factor model: *p* ≤ .0033; 8-factor model: *p* ≤ .0018) are shown.

^a^ Low numerical inaccuracy due to state-of-the-art approximations in the model estimation.

**Figure 1:**
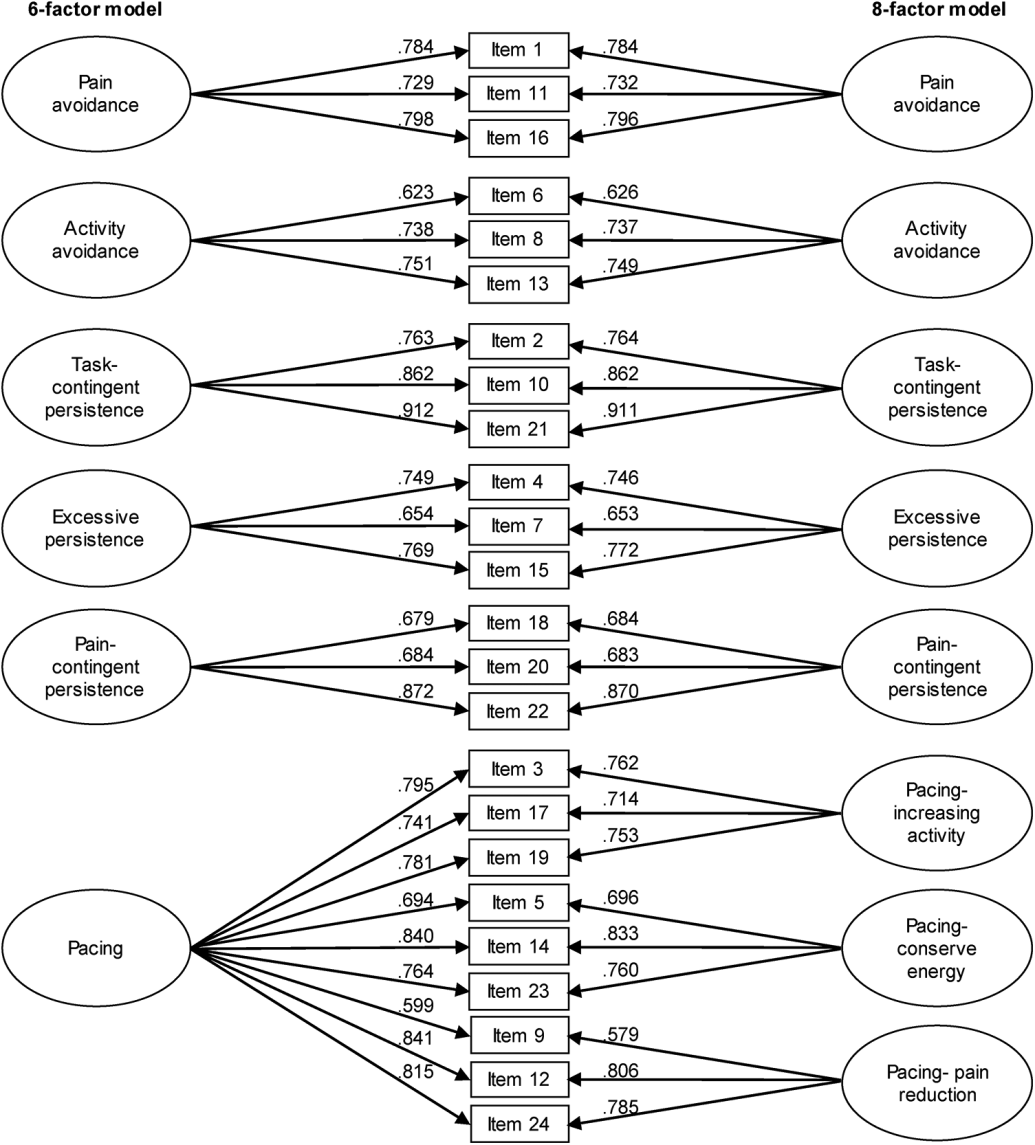
Confirmatory factor analysis of the 6- and 8-related factor solutions of the APS-GE. Decimal numbers positioned near arrows represent factor loadings on each subscale.

We employed confirmatory factor analysis to examine whether the factor structure described by Esteve et al. (5) could be reproduced by our data. Analogue to the original validation study, we tested three alternative factor structures: a three-factor (“avoidance”, “persistenc”e, and “pacing”), a six-factor (Figure 1) and an eight-factor model (Figure 1). For structural validation, non-orthogonal factor models were determined on ordinal items with robust diagonally weighted least squares (DWLS) model estimation and polychoric covariance determination. Correlations were calculated polychorically (factor models), according to Spearman (ordinal items) and Pearson (individual means). Standardized and case number independent goodness-of-fit indices such as the Comparative Fit Index (CFI) and the root mean square error of approximation (RMSEA) were used for this purpose (Table 7). The RMSEA, first described by Steiger et al. (42), defined as an absolute fit index, measures how far a hypothetical model deviates from a perfect model (43). A high value indicates a poor model fit. According to Hu et al. (44), the RMSEA should be ≤0.06. According to Browne et al. (45), RMSEA values in the range of 0.05 to 0.08 indicate a good fit. The CFI, on the other hand, compares a target model with an independent or null model. For a satisfactory fit, the value should be above 0.95 (44). Factor loadings of individual items are provided in Figure 1.

**Table 7:** Goodness-of-fit indeces resulting from confirmatory factor analysis of the APS-GE.

Alternative factor structures	CFI	RMSEA
Three factors	0.895	0.170
Six factors	0.968	0.096
Eight factors	0.974	0.088

CFI, comparative fit index; RMSEA, root mean square error of approximation.

##### 2.2.6.3 Construct and criterion validity- hypothesis testing

The evaluation of construct and criterion validity was based on the 8-factor solution of the APS-GE. To evaluate construct validity, in total, we formulated five *a priori* hypotheses describing expected relationships between the five APS-GE subscales addressing avoidance and persistence behavior with convergent or divergent constructs derived from validated instruments (sections *2.6.3 and 2.6.4*) (Table 8). Due to the unavailability of questionnaires assessing pacing behaviors in German language, no *a priori* hypotheses were formulated for the three pacing APS-GE subscales. Based on previous findings (5, 8), we hypothesized that the APS-GE subscales “pain avoidance”, “activity avoidance”, and “excessive persistence” would be positively associated with the cognitive processing style of catastrophizing (PCS) and activity or social avoidance assessed by FABQ and AEQ. At the same time, we predicted these APS-GE subscales to be inversely correlated with divergent constructs such as endurance behavior (AEQ). No significant correlations had been reported for APS subscales “task-contingent persistence” and “pain-contingent persistence” and catastrophizing when tested in patients with FMS (8) which informed the formulation of our hypotheses (Table 8). At the same time, we predicted the three APS-GE persistence subscales to be positively correlated with endurance behavior (AEQ).

**Table 8:** Construct validity (hypothesis testing): expected associations mainly based on the literature (5, 8) and theoretical considerations (left side of each cell) and observed Spearman's rank correlations between APS-GE subscales and convergent and divergent constructs (right side of each cell). Due to the lack of available data, *a priori* hypotheses could not be formulated for the APS-GE pacing subscales.

	Catas-trophizing (PCS)		Activity avoidance (FABQ)		Avoidance of physical activity (AEQ)		Avoidance of social activities (AEQ)		Endurance (AEQ)		Number of hypotheses met per subscale
Pain avoidance	+	.27*	+	.33*	+	.57*	+	.37*	−	−.20*	5/5 (100%)
Activity avoidance	+	.41*	+	.32*	+	.46*	+	.41*	−	−.03	4/5 (80%)
Task-contingent persistence	ns	−.03	-	−.13*	−	−.45*	−	−.28*	+	.29*	5/5 (100%)
Excessive persistence	+	.34*	+	.12*	+	−.02	+	.22*	+	.22*	4/5 (80%)
Pain-contingent persistence	ns	.22*	ns	.03	ns	.07	ns	.16*	+	.17*	3/5 (60%)
Pacing- increasing activity	?	.08	?	.21*	?	.37*	?	.14*	?	−.06	−
Pacing- conserve energy	?	.04	?	.13*	?	.39*	?	.13*	?	−.13*	−
Pacing- pain reduction	?	.19*	?	.31*	?	.51*	?	.24*	?	−.09	−
How many hypotheses were met in total?											**21/25 (84%)**

AEQ, avoidance-endurance questionnaire; FABQ, fear-avoidance beliefs questionnaire; PCS, pain catastrophizing scale; sample size for correlation analysis varies from *n* = 561 to *n* = 576 due to missing values; “+”: significant positive correlation expected; “−”: significant negative correlation expected; “ns”: non-significant correlation expected.

The green coloring indicates the agreement between hypotheses and empirical results.

* *p* < .005; significance level after Bonferroni correction per APS-GE subscale was set to *p* ≤ .01.

To evaluate criterion validity, German versions of questionnaires operationalizing outcomes relevant to chronic pain, such as pain intensity (CPGQ), impairment (CPGQ, PDI), and positive and negative affect (PANAS) were employed. For these outcomes, based on the results of the original validation study (5), six hypotheses were formulated per APS-GE subscale (Table 9). As suggested by Prinsen et al. (46), construct and criterion validity were considered satisfactory if ≥75% of the hypotheses per APS-GE subscale were confirmed.

**Table 9:** Criterion validity (hypothesis testing): expected associations based on Esteve et al. (5) (left side of each cell) and observed Spearman's rank correlations between APS-GE subscales and pain intensity, affect, and pain-related impairment (right side of each cell).

	Average pain intensity (CPGQ)^a^		Negative affect (PANAS)^a^		Positive affect (PANAS)^b^		Days of in-capacity for work (CPGQ)^a^^,^^#^		Impairment (CPGQ)^a^^,^^#^		Activity impairment (PDI)^a^		Number of hypotheses met per subscale
Pain avoidance	ns	.07	ns	.07	ns	−.12*	+	.26*	+	.22*	+	.23*	5/6 (83.3%)
Activity avoidance	ns	.28*	+	.26*	−	−.36*	+	.50*	+	.55*	+	.54*	5/6 (83.3%)
Task-contingent persistence	ns	.01	−	.07	+	.06	−	−.20*	−	−.16*	−	−.16*	4/6 (66.7%)
Excessive persistence	ns	.19*	+	.39*	ns	−.24*	+	.23*	+	.27*	+	.31*	4/6 (66.7%)
Pain-contingent persistence	ns	.05	ns	.08	ns	.00	ns	.10	ns	.12*	ns	.11*	4/6 (66.7%)
Pacing- increasing activity	ns	.10*	ns	−.08	+	.02	−	.22*	ns	.22*	ns	.19*	1/6 (16.7%)
Pacing- conserve energy	ns	.06	−	−.13*	+	.09	−	.19*	ns	.15*	ns	.12*	2/6 (33.3%)
Pacing- pain reduction	ns	.19*	ns	.00	ns	−.07	+	.31*	+	.30*	+	.30*	5/6 (83.3%)
How many hypotheses were met in total?													**30/48** (**62.5%)**

CPGQ, chronic pain grade questionnaire; PDI, pain disability Inventory; sample size for correlation analysis varies from *n* = 567 to *n* = 578 due to missing values.

The green coloring indicates the agreement between hypotheses and empirical results.

^a^ Higher scores correspond to worse outcome/ higher symptom severity.

^b^ Higher scores correspond to better outcome/ lower symptom severity.

^#^ Ratings refer to the last 3 months; “+”: significant positive correlation expected; “−”: significant negative correlation expected; “ns”: non-significant correlation expected;.

* *p* < .005; significance level after Bonferroni correction per APS-GE subscale was set to *p* ≤ .008 per APS-GE subscale.

We additionally analyzed associations of APS-GE subscales with psychological distress (DASS) and health-related Qol (VR-12) (Table 10). Correlational analysis for the assessment of construct and criterion validity used Spearman's rank correlation as APS-GE subscale scores could not be considered metrically scaled.

**Table 10:** Criterion validity (explorative approach): spearman's rank correlations between APS-GE subscales and additional outcomes of high relevance to chronic pain (psychological distress and health-related quality of life). *a priori* hypotheses could not be formulated due to the lack of existing data.

	Depression (DASS)^a^	Anxiety (DASS)^a^	Stress (DASS)^a^	Physical Qol (VR-12)^b^	Mental Qol (VR-12)^b^
Pain avoidance	.18*	.09	.11*	−.19*	−.11
Activity avoidance	.42*	.26*	.28*	−.53*	−.23*
Task-contingent persistence	−.05	.02	.04	.19*	.03
Excessive persistence	.36*	.32*	.44*	−.15*	−.30*
Pain-contingent persistence	.12*	.14*	.15*	−.06	−.08
Pacing- increasing activity	.00	.13*	−.07	−.31*	.06
Pacing- conserve energy	−.06	.06	−.11*	−.26*	.12*
Pacing- pain reduction	.09	.13*	.00	−.40	.00

^a^ Higher scores correspond to worse outcome/ higher symptom severity.

^b^ Higher scores correspond to better outcome/ lower symptom severity; the strength of positive association between two variables is illustrated by a gradient of shades of blue; the strength of negative association between two variables is illustrated by a gradient of shades of red.

* Significance level after Bonferroni correction per APS-GE subscale was set to *p* ≤ .007.

DASS, depression anxiety stress scale; VR-12, veterans rand 12-Item health survey; Qol, quality of life; sample size for correlation analysis varies between *n* = 577 and *n* = 578 due to missing values.

##### 2.2.6.4 Criterion validity- discriminatory potential

Discriminatory power of APS-GE subscales was evaluated comparing individuals with and without prior exposure to psychotherapeutic, psychiatric or interdisciplinary, multimodal pain treatment with respect to the distribution of APS-GE activity patterns. For these analyses, we chose a non-parametric approach by Mann–Whitney *U* or Kruskal–Wallis tests because prerequisites for a parametric approach (e.g., normal distribution) were violated (Table 11).

**Table 11:** Discriminatory power of APS-GE subscales with respect to gender and exposure to pre-treatment.

	Pain avoidance	Activity avoidance	Task-contingent persis-tence	Excessive persis-tence	Pain-contingent persis-tence	Pacing-increasing activity	Pacing-conserve energy	Pacing-pain reduction
Gender identity (male vs. female)	Z = −2.404 (***p* < .001**)	Z = −.864 (*p* = .387)	Z = −.040 (*p* = .968)	Z = 1.945 (*p* = .052)	Z = 1.061 (*p* = .288)	Z = −.981 (*p* = .327)	Z = −.859 (*p* = .390)	Z = −2.249 *(p* *=* *.025)*
Exposure to pre-treatment (no vs. yes)								
Outpatient psychotherapy	Z = −.120 (*p* = .905)	Z = 3.251 (***p* < .001**)	Z = −.944 (*p* = .345)	Z = 3.195 (***p* < .001**)	Z = .144 (*p* = .885)	Z = 2.019 *(p* *=* *.043)*	Z = 1.021 (*p* = .307)	Z = 1.256 (*p* = .209)
Psychiatric treatment^a^	Z = −.449 (*p* = .653)	Z = 3.140 (***p* = .002**)	Z = −1.273 (*p* = .203)	Z = 3.601 (***p* < .001**)	Z = −.222 (*p* = .824)	Z = .520 (*p* = .603)	Z = .363 (*p* = .716)	Z = .570 (*p* = .569)
Interdisciplinary, multimodal pain therapy^a^	Z = −1.433 (*p* = .152)	Z = 1.561 (*p* = .118)	Z = .071 (*p* = .944)	Z = 2.268 *(p* *=* *.023)*	Z = −.842 (*p* = .400)	Z = −.343 (*p* = .731)	Z = .079 (*p* = .937)	Z = .626 (*p* = .531)

Sample sizes vary due to missing data; APS-GE, German version of the Activity Patterns Scale; group comparisons by Mann–Whitney *U* or Kruskal–Wallis tests, significance level after Bonferroni correction:.05/5 = *p* ≤ .01, statistically significant *p*-values in bold, nominally significant *p*-values in italics.

^a^ Different settings included.

##### 2.2.6.5 Criterion validity- hierarchical multiple regression analysis

To explore the relative contributions of APS-GE activity patterns and comparable constructs derived from existing instruments (PCS, FABQ, and AEQ) to the explanation of variance in two different pain-relevant outcome domains (disability/ functional impairment and psychological distress), two hierarchical multiple regression analyses were performed. We calculated a disability composite score representing the average of the z-standardized PDI and CPGQ disability sum scores. A psychological distress composite score was calculated from the following z-standardized scores: DASS depression, DASS anxiety and DASS stress, and PANAS negative affect. Variables individually significantly associated with the disability composite score were selected as predictors. We entered these into the model in six steps to determine R^2^ change (increase in explained variance by each step). We started with basic sociodemographic and clinical variables (steps 1 and 2), followed by catastrophizing (PCS, step 3), activity avoidance (FABQ, step 4), and avoidance of physical and social activities and endurance (AEQ, step 5), ending with the APS-GE subscales (step 6, Supplementary Material S3). The same set of predictor variables and procedure was used for the hierarchical multiple regression analyses using the psychological distress composite score for comparability purposes (Supplementary Material S4).

## 3 Results

### 3.1 Sociodemographic and clinical sample description

In total, the psychometric evaluation of the APS-GE was based on data sets of 579 patients with primary or secondary musculoskeletal pain (refer to Table 1 for detailed sociodemographic and Tables 2, 3 for basic clinical information). The distribution among the clinical subgroups was as follows (ordered by ascending percentage): RA and FMS (2.9%), upper and lower extremity pain (9.6%), RA and other inflammatory diseases (11.7%), multisite pain (14.2%), FMS (19.7%), and non-specific neck and back pain (41.5%). A total of 74.8% of participants identified as women and 25.2% as men. On average, participants were 53.8 years (±12.2 SD) old and 73.4% were in a relationship or married. The majority had some kind of school-leaving qualification ranging from low primary education (22.3%) to general high school education (23.1%). Fifty-four percent of participants were in full- or part-time employment, 31% were retired and the remaining 15% were either unemployed or incapacitated for work. The average duration of pain was 146.8 months (±126 SD). Fibromyalgia patients had a significantly higher widespread pain index and somatic symptom severity than all other clinical subgroups. Individuals with multisite pain outscored participants with upper and lower extremity pain on the widespread pain index. About fifty-eight percent of the sample were on continuous analgesic medication and 74.5% reported to experience pain relief from their analgesic drug regimen. Regarding the type of oral analgesics, 70.4% were on non-opioids, 34.8% on opioids, 18.8% on antidepressants, 15.4% on anticonvulsants and 6.6% on other pain medication. Most of the participants received oral analgesic monotherapy (46%) or a combination of two analgesic drug classes (28.4%). About half of the study sample (48.5%) had not received any kind of psychiatric pre-treatment, 36% had received psychiatric outpatient care and 15.6% had undergone inpatient psychiatric treatment. About 45% of participants had never had regular psychotherapy sessions or interdisciplinary multimodal pain therapy of any setting. For differences between clinical subgroups with respect to the distribution of sociodemographic and clinical baseline variables we refer to Tables 1–3.

### 3.2 Reliability

Internal consistency of APS-GE subscales (Table 4) ranged between Cronbach's α = 0.71 (activity avoidance, excessive persistence) and Cronbach's α = 0.84 (task-contingent persistence). Analyses of test-retest reliability were based on *n* = 52 complete data sets. The average test-retest time interval was 16.2 days (±2.9 SD; min. 7/max. 24). Table 5 shows the test-retest reliability statistics of APS-GE subscales and single items. Moderate test-retest reliability resulted for all subscales with ICC_2,1_ ranging from 0.50 (*task-contingent persistence*) to 0.71 (*pain avoidance*, *pacing-increasing activity*). All test-retest correlations for single items were statistically significant, except for item 2 (*r* = 0.25; “Kept on doing what I was doing”). We obtained highest stability estimates for item 8 belonging to the activity avoidance subscale (*r* = 0.78; “Because of my pain most days I spend more time resting than doing activities”).

### 3.3 Structural validity

An item and scale analysis of the APS-GE (Table 4) revealed acceptable corrected item-factor correlations ranging from 0.44 to 0.76. We performed a confirmatory factor analysis to evaluate the reproducibility of a three-factor, a six-factor and an eight-factor model as proposed by Esteve et al. (5). For the three-factor solution, items were assigned to the following factors: avoidance (items 1, 6, 8, 11, 16), persistence (items 2, 4, 7, 10, 15, 18, 20, 21, 22), and pacing (items 3, 5, 9, 12, 14, 17, 19, 23, 24). For the item assignment of the six- and eight-factor solutions, we refer to Figure 1. Based on our data, the original factor structure could be reproduced very well. As can be seen in Table 7, the three-factor model failed to meet the recommended cut-off criteria. The model fit was satisfactory for both the 6- and 8-factor solutions, with CFI of 0.968 and 0.974 and RMSEA of 0.096 and 0.088, respectively (Table 7). As expected, all items showed significant positive factor loadings (*p* < 0.0005), with standardized coefficients ranging from 0.626 to 0.912 (Figure 1). As demonstrated in Table 6, high covariance estimates resulted for the APS-GE pacing subscales varying around Spearman's rank correlation coefficients of 1. *Pain avoidance* was moderately positively correlated with *activity avoidance* (rho = 0.64) and the three pacing subscales (rho = 0.44 to 0.63) with highest covariance estimates for *pacing- pain reduction*. Although *pain avoidance* was not correlated with *excessive persistence* and *pain-contingent persistence*, it was negatively correlated with *task-contingent persistence* (rho = −0.51). Unexpectedly, a moderate positive correlation was found between *activity avoidance* and *excessive persistence* (rho = 0.43). *Activity avoidance* was further positively associated with the pacing subscales with highest estimates for *pacing- pain reduction* (rho = 0.68). Like *pain avoidance*, *activity avoidance* was negatively correlated with *task-contingent persistence* (rho = −0.37). The three persistence subscales were moderately correlated with each other (rho = 0.29 to 0.41). *Excessive persistence* had low but significant negative correlations with the pacing subscales (rho = −0.14 to −0.19). *Task-contingent persistence* was moderately inversely correlated with the three pacing subscales (rho = −0.39 to −0.53). For *pain-contingent persistence*, a low positive correlation was observed with *pacing- conserve energy* (rho = 0.16), while for the remaining pacing subscales correlations with *pain-contingent persistence* were not statistically significant.

### 3.4 Construct validity- hypothesis testing

For each of the eight APS-GE subscales (except the three pacing subscales), five hypotheses reflecting the expected association with convergent and divergent constructs assessed by established questionnaires were formulated (Table 8). As predicted, APS-GE pain avoidance and activity avoidance correlated positively with convergent constructs assessed by established instruments. APS-GE pain avoidance showed a significant negative association with AEQ endurance while APS-GE activity avoidance did not. In line with our hypotheses, APS-GE task-contingent persistence was not linked to PCS catastrophizing and positively correlated with AEQ endurance. Expectedly, APS-GE task-contingent persistence was negatively correlated with FABQ activity avoidance, AEQ avoidance of physical activity, and AEQ avoidance of social activities. APS-GE excessive persistence was significantly associated with catastrophizing (PCS), activity avoidance (FABQ), avoidance of social activities (AEQ) and endurance (AEQ). Of all three APS-GE persistence subscales, pain-contingent persistence showed the lowest positive association with AEQ endurance. It was significantly associated with PCS catastrophizing and had a weak association with AEQ avoidance of social activities. The three APS-GE pacing subscales showed positive correlations with the questionnaire subscales assessing avoidance while only pacing- pain reduction was positively associated with catastrophizing (PCS). Only APS-GE pacing- conserve energy had a mild negative association with AEQ endurance, however. According to the criteria suggested by Prinsen et al. (46), except for *pain-contingent persistence*, construct validity can be considered satisfactory for all APS-GE subscales (confirmation of ≥75% of hypotheses). Averaged across all APS-GE subscales, 84% of hypotheses could be confirmed.

### 3.5 Criterion validity- hypothesis testing

To evaluate criterion validity, hypotheses about associations of the eight APS-GE subscales with pain intensity, negative affect, positive affect, days of incapacity for work, and activity impairment were formulated and tested. As shown in Table 9, the higher the level of *pain avoidance*, the lower the positive affect, the more days of incapacity for work in the past three months, and the higher the activity impairment. Overall, correlations for *pain avoidance* with the criteria selected were low to moderate. For *activity avoidance*, in contrast, moderate to high positive correlations were detected for pain intensity, negative affect, days of incapacity for work, and activity impairment. A moderate negative correlation was found for *activity avoidance* and negative affect. Consistent with our expectations, low to moderate negative correlations resulted for *task-contingent persistence* and days of incapacity for work, and activity impairment. The higher the level of *excessive persistence*, the higher the average pain intensity, the negative affect, the number of days of incapacity for work, and the level of impairment and the lower the positive affect. *Pain-contingent persistence* was only weakly positively associated with activity impairment. All three pacing subscales were positively associated with days of incapacity for work, and activity impairment with *pacing- pain reduction* showing the strongest link. Both *pacing- increasing activity* and *pacing- pain reduction* showed low positive associations with pain intensity while *pacing- conserve energy* did not. Overall, for *pacing- pain reduction*, a correlation pattern emerged resembling the one of the subscale *pain avoidance*. *Pacing- conserve energy* was the only APS-GE subscale inversely correlated with negative affect. Averaged across all APS-GE subscales, 62.5% of hypotheses could be confirmed by our data, ranging from 83.3% for *pain avoidance*, *activity avoidance*, and *pacing- pain reduction* to 16.7% for *pacing- increasing activity*.

Additionally, correlations of the eight APS-GE subscales with depression, anxiety, stress, and physical and mental quality of life (Qol) were explored. *Activity avoidance* and *excessive persistence* showed the highest positive correlations with depression, anxiety and stress and negative association with physical and mental Qol. *Task-contingent persistence* was found to be positively associated with physical Qol while not being associated with depression, anxiety, stress, and mental Qol. *Pain-contingent persistence* was weakly positively correlated to depression, anxiety, and stress. *Pacing- conserve energy* was the only APS-GE subscale inversely correlated with stress and physical Qol and positively correlated with mental Qol while the other two pacing subscales showed weak positive correlations with anxiety and moderate negative associations with physical Qol.

#### 3.5.1 Criterion validity- discriminatory potential of APS-GE dimensions

Interestingly, female study participants had higher scores on the pain avoidance dimensions as compared to male participants (*Z* = −2.404, *p* < 0.001, Table 11). The remaining APS-GE dimensions were equally distributed among genders. Importantly, individuals who had been exposed to outpatient psychotherapy or psychiatric treatment of different settings showed significantly lower *activity avoidance* (*Z* = 3.251, *p* < 0.001 and *Z* = 3.140, *p* = 0.002, respectively) and *excessive persistence* (*Z* = 3.195, *p* < 0.001 and *Z* = 3.601, *p* < 0.001, respectively). A nominally significant improvement in *pacing for the purpose to increasing the activity level* could be seen in individuals who had received outpatient psychotherapy (*Z* = 2.019, *p* = 0.043). Exposure to interdisciplinary, multimodal pain therapy was not associated with any differences in the distribution of activity patterns, except for a nominally significant effect for *excessive persistence* (*Z* = 2.268, *p* = 0.023).

#### 3.5.2 Criterion validity- hierarchical multiple regression analysis

To evaluate the relative predictive impact of the APS-GE subscales and established instruments measuring pain-related activity patterns on disability/ impairment and psychological distress, two hierarchical multiple regression analyses were performed.

##### 3.5.2.1 Disability composite score as dependent variable

Basic sociodemographic variables found to significantly correlate with the disability composite score were included as confounders in the first step of the model. Here, the highest school-leaving qualification appeared as a significant predictor of the disability composite score. As a next step, clinical variables individually associated with the disability composite score were added and the widespread pain index and the somatic symptom severity emerged as significant contributors to the explanation of variance in the predictor (*R*^2^_change_ = 0.229, *p* < 0.001). Third, catastrophizing (PCS) was added which led to a large increase in the explanation of variance of *R*^2^_change_ = 0.151 (*p* < 0.001). At step 4, activity avoidance (FABQ) was entered into the model which led to only a mild increase in the explanation of variance (*R*^2^_change_ = 0.012, *p* = 0.001). Next, the subscale scores of the AEQ were added, which again only mildly contributed to the model (*R*^2^_change_ = 0.019, *p* < 0.001). At this step, only AEQ avoidance of social activity appeared as independent predictor (*β* = 0.115, *p* = 0.007) of the disability composite score in addition to the variables already added to the model at the previous steps. At the last step, the eight APS-GE subscale scores were included which led to a change in *R*^2^ of 0.102 (*p* < 0.001). Upon inclusion of APS-GE activity avoidance (*β* = 0.356, *p* < 0.001), FABQ activity avoidance no longer contributed to the model. The final model explaining 53.8% of variance in the disability composite score included the following independent predictors (sorted by ascending magnitude of regression coefficient): avoidance of social activities (AEQ), highest school-leaving qualification, widespread pain index, somatic symptom severity, catastrophizing (PCS), activity avoidance (APS-GE) (Supplementary Material S3).

##### 3.5.2.2 Psychological distress composite score as dependent variable

Psychological distress is an outcome domain of high relevance to chronic pain syndromes. Therefore, we applied the methodological approach outlined above to a psychological distress composite score. At step 1, the highest school-leaving qualification appeared as a significant predictor of psychological distress. At step 2, the clinical variables were included. In contrast to the above model, only somatic symptom severity emerged as a significant predictor of psychological distress which contributed substantially to the explanation of variance (*R*^2^_change_ = 0.394, *p* < 0.001). Highest school-leaving qualification did no longer contribute to the explanation of variance at this step. Catastrophizing (PCS) was entered next and led to a substantial increase in the explanation of variance of *R*^2^_change_ = 0.169 (*p* < 0.001). FABQ activity avoidance (step 4) did not contribute significantly to this model. At step 5, both avoidance of physical and social activities (AEQ) appeared as significant predictors of psychological distress. The increase in explanation of variance was minor, however (*R*^2^_change_ = 0.014, *p* < 0.001). At step 6, both APS-GE excessive persistence and pacing- conserve energy emerged as independent predictors with a larger effect for *excessive persistence*. The final model explaining 59.8% of variance in the psychological distress composite score contained the following predictors (sorted by ascending magnitude of regression coefficient): excessive persistence (APS-GE), avoidance of physical activities (AEQ), avoidance of social activities (AEQ), somatic symptom severity, and catastrophizing (PCS) (Supplementary Material S4).

## 4 Discussion

The present study aimed at culturally adapting the activity pattern scale for scientific and clinical use in German-speaking countries. A sample of 579 patients presenting with musculoskeletal pain was recruited to perform a psychometric evaluation of the German version. Confirmatory factor analysis of APS-GE items reproduced the six and eight related factor structure proposed by Esteve et al. (5) supporting the multidimensionality of avoidance and persistence behavior. In conjunction with the validity analyses, on the basis of our data and previous studies (5, 47), we strongly recommended to clinically differentiate between task-contingent persistence and excessive overactivity. Moreover, we confirmed our hypotheses regarding the internal consistency of APS-GE subscales and the corrected item-factor correlations of individual items. Our findings in the context of the construct validity of APS-GE subscales mostly agreed with the literature.

Our study complements the existing literature in several ways: First, it adds to the preliminary data on the temporal stability of pain-related activity patterns (48). Second, it offers a very detailed clinical description of a heterogeneous study sample. The assessment of the representativeness of the sample has important implications for the external validity of our results and the interpretation of psychometric indices such as Cronbach's α (39). Moreover, to our knowledge, this is the first study to analyze the relative predictive value of different questionnaires operationalizing analogous constructs while controlling for possible confounders. Our findings underline the clinical utility of the APS-GE in comparison to other instruments. Deviations from the results of previous investigations regarding the construct and criterion validity of APS-GE subscales are discussed below.

### 4.1 Varying temporal stability of APS-GE items

To our knowledge, this is the second study to address the test-retest reliability of APS-GE dimensions over a time interval of two weeks. We expected pain-related activity patterns to oscillate over time due to modulatory influences of current goal hierarchies, and other personal or external factors (49). Consistent with our predictions, moderate test-retest reliabilities resulted for all subscales indicating their relative change sensitivity. As we predicted on theoretical grounds, we found temporal stability to vary across APS-GE subscales suggesting that some activity patterns are more context-sensitive than others. Our findings contradict the results of a recent longitudinal study using the APS showing no linear change in activity patterns (rated each day) over a 15-day period, except for *excessive persistence* (3). Whether variations over time follow nonlinear trends or vary depending on clinical and motivational variables remains to be clarified by future studies.

Recently, a German version of the APS has been published which was developed in the German-speaking part of Switzerland and evaluated in 65 individuals suffering from chronic musculoskeletal pain (48). At the start of our investigation, a German version of the APS was not yet available. In line with the findings of Hotz-Boendermaker et al. (48), both APS-GE dimensions of avoidance behavior displayed a high degree of temporal stability. Our findings are in line with psychometric studies reporting moderate to high test-retest reliability for instruments assessing catastrophizing (50) or fear-avoidance behavior (51). The high temporal stability could be explained by the “overgeneralization” of avoidance behavior through mechanisms of classical conditioning in individuals with chronic pain (52). Interestingly, consistent with a previous psychometric study (48), lowest test-retest stability was found for *task-contingent persistence* suggesting that this activity pattern may be highly susceptible to contextual and motivational factors. Whether someone completes a task despite an increase in pain, may depend in part on the nature of the task and its function with respect to the achievement of currently prioritized goals. Supporting this, experimental studies converge on the observation that when faced with a valued goal (e.g., monetary reward), study participants are more likely to carry on with an activity and avoidance behavior is attenuated (49).

### 4.2 Construct and criterion analysis reveals conceptual ambiguities

Regarding the construct validity of *pain avoidance*, all hypotheses based on the existing literature could be confirmed. Contrary to our predictions, *activity avoidance* failed to be inversely associated with endurance (AEQ). This may be partly explained by AEQ endurance capturing cognitive and behavioral responses to pain episodes while the APS-GE activity avoidance subscale addresses more general changes in activity level without referring to fluctuations in pain intensity. A general decrease in activity level may also reflect a lack of drive associated with depressive symptoms which are highly prevalent in chronic pain (53). This notion is substantiated by our data. Relative to the other APS-GE subscales, *activity avoidance* was most closely linked to high pain intensity, high negative affect, reduced positive affect, high activity impairment, depression, anxiety, stress, and reduced physical and mental Qol. In addition, APS activity avoidance was previously associated with motivational constructs highly prevalent in depressed individuals, such as pessimism, and reduced self-efficacy (47). Thus, our data contribute to the existing literature by replicating the maladaptivity of avoidance behavior. Potential conceptual overlaps between some items of APS-GE activity avoidance and the depression symptom dimension “lack of drive” should be addressed by future studies.

All hypotheses regarding the construct validity of *task-contingent persistence* could be confirmed. In line with the findings of others (5), our data also show that *task-contingent persistence* has beneficial effects on daily functioning and physical Qol. In contrast to previous work, in our German sample, *task-contingent persistence* was not associated with mental wellbeing or reduced psychological distress. Besides differences in the operationalization of outcomes, this interesting discrepancy could be partly attributed to intercultural differences in distinct dimensions of achievement motivation (54). Accounts which integrate a cultural perspective into achievement motivation theory (55) argue that the perceived value of “achievement” varies cross-culturally (56). Recent evidence suggests that Germany has a more individualistic value orientation than Spain emphasizing effort, intrinsic motivation, and willpower (57). Consequently, Germans may be more inclined to regulate their self-esteem through individual achievement and to attribute “failure” to personal shortcomings rather than contextual factors. This could predispose to a more avoidance-oriented form of achievement motivation that aims at preventing loss of self-esteem. Interestingly, *excessive persistence* covaried to a similar extent with AEQ endurance and established measures of catastrophizing and avoidance behavior suggesting that excessive forms of overactivity may be motivated by avoidance goals (e.g., avoidance of loss of self-esteem) in some individuals. In support of this view, preliminary evidence (8) relates all three APS-GE persistence subscales to a maladaptive dimension of perfectionism addressing e.g., “concerns over mistakes” and “excessively high personal standards” (58). Highest associations were found for this avoidance-oriented form of perfectionism with *excessive persistence*. In accordance with other authors, we found all pacing subscales to be positively correlated with measures of avoidance behavior. Moreover, associations among the three pacing subscales were very high suggesting partial conceptual congruence which, viewed in isolation, calls into question the multidimensionality of activity pacing. In contrast, the usefulness of differentiating between pacing behaviors based on underlying goals becomes particularly evident when evaluating correlations with clinical outcome parameters, however.

### 4.3 Clinical implications

When simultaneously controlling for confounders and existing measures of pain-related activity patterns, APS-GE activity avoidance emerged as the strongest predictor of disability underlining the clinical usefulness of the APS-GE. Interestingly, we found *activity avoidance* and *excessive persistence* to be less pronounced in individuals with prior exposure to psychotherapeutic or psychiatric pre-treatment. This may be interpreted as a first hint towards treatment responsiveness of the behavioral constructs measured with the APS-GE.

In summary, we join others in assuming that the adaptivity of a certain activity pattern may be significantly influenced by motivational factors (59, 60). Supported by empirical findings, the type of goal (approach vs. avoidance) prioritized in a situation at hand seems to play a pivotal role with respect to feelings of impairment, or psychological distress (61). In line with two-factor models such as Gray's reinforcement sensitivity theory (RST) (62) and the BIS-BAS model of pain (63), we found APS-GE subscales operationalizing avoidance behaviors across all superordinate dimensions (i.e., *activity avoidance*, *excessive persistence*, and *pacing for the purpose of pain reduction*) to be associated with cognitive-affective processes and outcomes compatible with the functional profile of a behavioral inhibition system (BIS) (63). *Pacing for the purpose of conserving energy for valued activities* can be considered the only behavioral dimension of the APS explicitly linked to a rewarding goal (i.e., the pursuit of valued activities). Consistent with the functional profile of the Behavioral Activation System mediating approach behavior motivated by anticipated reward (63), *Pacing- conserve energy* appeared to be most beneficial with respect to psychological variables while associations with functional impairment were rather low. Consequently, *pacing- conserve energy* may reflect an adaptive way of pain management by balancing the pursuit of nonpain-related and pain-related goals.

Current state-of-the-art pain management programs involve the teaching of different pacing strategies such as the pain-independent interruption of tasks by short recovery breaks (21). In line with previous work, our findings emphasize the importance of explicitly addressing the goals motivating the use of certain pacing strategies. Therapeutic approaches should encourage the orientation towards nonpain-related, self-determined goals (64). Preliminary evidence indicates that the relationship between activity pacing and avoidance varies as a function of a person's exposure to treatment (65). These findings imply that during therapy, spontaneous pacing strategies largely aiming at pain control are replaced by planned, seemingly counter-intuitive pacing strategies aiming at “functional restoration” or “energy conservation” (7).

### 4.4 Limitations

Our findings must be interpreted in the light of several limitations. Firstly, the assessment of behavioral constructs via self-report instruments may merely reflect an individual's belief about the frequency of certain behaviors and their introspective abilities. In the future, construct validity of activity patterns should be tested using additional psychophysiological and behavioral measures. Using a 5-day observational design, Andrews et al. (66) offer some preliminary evidence for a high association between objectively measured levels of activity via an activity monitor and self-reported levels of overactivity.

In reality, individuals may use various combinations of activity patterns to adjust to a life with persistent pain. In this study, we did not evaluate the relationships of empirically confirmed clusters of activity patterns with functioning, which limits the external validity of our result (65). Moreover, the correlative nature of the results does not allow any causal statements to be made. Here, more longitudinal and experimental studies are needed to strengthen our understanding of causal mechanisms. Regarding methodological shortcomings of our study, we fell short of the planned sample size for the test-retest reliability analysis, so that our sample may be slightly underpowered to detect ICCs of 0.6 to 0.8. Additionally, the hierarchical multiple linear regression analyses are to be interpreted with caution, as we did not simultaneously analyze residual plots to avoid biases (67).

## 5 Conclusions

In general, the psychometric properties of the APS-GE appear to be acceptable making it a promising instrument for diagnostics and the monitoring of therapeutic progress. Our data contribute to the existing literature by replicating the maladaptivity of avoidance behavior, excessive persistence and pain-contingent pain management behaviors. In our German sample, *task-contingent persistence* appeared to be beneficial with respect to functional outcomes, while *pacing for the purpose of energy conservation* was related to positive psychological outcomes. Besides methodological issues, inconsistencies to previous studies may be due to intercultural differences in different dimensions of achievement motivation which may vary along the individualism-collectivism continuum which may open up a new field of research. The extent to which our findings regarding the test-retest reliability of the different behavioral dimensions generalize to other populations warrants further investigation. Given the conceptual ambiguities discussed above, content validity of some APS-GE subscales needs to be reconsidered. To improve clinical and scientific utility, especially for research conducted from a self-regulation perspective, the linking of activity patterns with approach and avoidance goals should be implemented more consistently across all APS-GE subscales.

## Data Availability

The raw data supporting the conclusions of this article will be made available by the authors, without undue reservation.
