# Supplementary material for: Cross-cultural adaptation and psychometric evaluation of a German version of the Activity Patterns Scale (APS-GE) in a large sample of patients with chronic musculoskeletal pain
Source: Front Pain Res (Lausanne). 2025 Jun 13;6:1570432. doi: 10.3389/fpain.2025.1570432 (PMC12202367; doi:10.3389/fpain.2025.1570432)
Supplement: Supplementary file 1 [file Table2.docx]

**Cross-cultural adaptation and** **psychometric evaluation of a German version of the Activity Pattern Scale (APS-GE) in a large sample of patients with chronic musculoskeletal pain**

**Anne Kästner^1^, Margarete Donhauser^1^****, Inga von Freytag-Löringhoff^1^, Frank Petzke^1^**

**^1^Department of Anesthesiology, Pain Clinic, University Hospital, Georg-August-University of Göttingen, Germany**

**Supplementary Material 2: German version of the Activity Pattern Scale (APS-GE)**


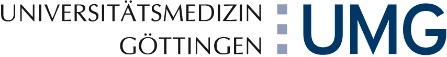


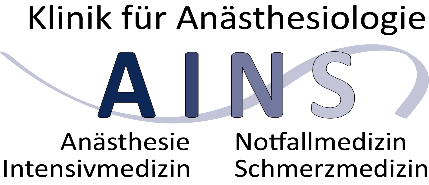


| **Fragebogen zu Aktivitätsmustern bei chronischen Schmerzen (APS-GE)** |
| --- |

Personen, die Schmerz empfinden, führen ihre täglichen Aktivitäten unterschiedlich aus. Bitte überlegen Sie, wie Sie normalerweise Ihre täglichen Routinen durchführen und geben Sie an, ob die Aussagen das wiederspiegeln, was Sie gewöhnlich tun. Dabei bedeutet 0 „niemals“ und 4 „immer“.

Nehmen Sie z.B. die Aussage „Ich unterbreche eine Aktivität sofort, wenn meine Schmerzen schlimmer werden.“ Wenn dieser Satz eine Verhaltensweise beschreibt, die Sie im Alltag sehr oft an den Tag legen, wählen Sie 4 (immer) oder 3 (häufig). Wenn Sie ein Verhalten „niemals“ oder „selten“ zeigen, dann kreuzen Sie 0 oder 1 an. Wenn Sie etwas „manchmal“ machen, wählen Sie die 2.

**Einige Aussagen ähneln sich zwar, sind aber nicht identisch. Daher lesen Sie bitte alle Aussagen aufmerksam und setzen Sie in jeder Reihe ein Kreuz.**

|  | **Niemals**  **0** | **Selten**  **1** | **Manch-mal**  **2** | **Häufig**  **3** | **Immer**  **4** |
| --- | --- | --- | --- | --- | --- |
| 1. Ich unterbreche eine Aktivität sofort, wenn meine Schmerzen anfangen schlimmer zu werden. | ( ) | ( ) | ( ) | ( ) | ( ) |
| 1. Ich führe Aufgaben so lange aus, bis ich sie beendet habe. | ( ) | ( ) | ( ) | ( ) | ( ) |
| 1. Gewöhnlich mache ich mehrere Ruhepausen und so kann ich viel mehr erledigen. | ( ) | ( ) | ( ) | ( ) | ( ) |
| 1. Ich versuche zu viel zu erledigen und fühle mich in der Folge (danach) schlechter. | ( ) | ( ) | ( ) | ( ) | ( ) |
| 1. Ich teile Aktivitäten in kleine Abschnitte ein und kann so Energie für andere Dinge übrigbehalten, die mir wichtig sind. | ( ) | ( ) | ( ) | ( ) | ( ) |
| 1. Ich bin nicht mehr in der Lage mit meinem gewöhnlichen Aktivitätsniveau weiterzumachen. | ( ) | ( ) | ( ) | ( ) | ( ) |
| 1. Ich beeile mich immer sehr, alles erledigt zu haben, bevor ich nicht mehr kann. | ( ) | ( ) | ( ) | ( ) | ( ) |
| 1. Wegen meiner Schmerzen verbringe ich die meiste Zeit des Tages mit Ausruhen, statt Aktivitäten nachzugehen. | ( ) | ( ) | ( ) | ( ) | ( ) |
| 1. Ich mache Dinge langsamer, damit es weniger weh tut. | ( ) | ( ) | ( ) | ( ) | ( ) |
| 1. Wenn ich mit einer Tätigkeit beginne, höre ich nicht auf, bevor sie wirklich erledigt ist. | ( ) | ( ) | ( ) | ( ) | ( ) |
| 1. Wenn ich weiß, dass eine Aktivität meine Schmerzen verschlimmert, mache ich sie nicht mehr. | ( ) | ( ) | ( ) | ( ) | ( ) |
| 1. Ich teile Aktivitäten in kleine Abschnitte ein, damit ich weniger Schmerzen habe. | ( ) | ( ) | ( ) | ( ) | ( ) |
| 1. Manche Aktivitäten, die zu meinem Leben gehören, muss ich ruhen lassen. | ( ) | ( ) | ( ) | ( ) | ( ) |
| 1. Gewöhnlich mache ich mehrere Ruhepausen. So kann ich Energie sparen, um Dinge zu tun, die mir wichtig sind. | ( ) | ( ) | ( ) | ( ) | ( ) |
| 1. Ich übernehme mich und mache zu viele Dinge auf einmal, und dann muss ich für eine Weile ausruhen. | ( ) | ( ) | ( ) | ( ) | ( ) |
| 1. Ich vermeide es, Dinge zu machen, die Schmerzen verursachen. | ( ) | ( ) | ( ) | ( ) | ( ) |
| 1. Ich mache Dinge langsamer, um so viel mehr schaffen zu können. | ( ) | ( ) | ( ) | ( ) | ( ) |
| 1. Wenn ich wenig Schmerzen habe, versuche ich so aktiv zu sein, wie es nur geht. | ( ) | ( ) | ( ) | ( ) | ( ) |
| 1. Ich unterteile Aktivitäten in kleine Abschnitte und kann auf diese Weise viel mehr schaffen. | ( ) | ( ) | ( ) | ( ) | ( ) |
| 1. Ich mache viel mehr Dinge als normalerweise, wenn ich weniger Schmerzen habe. | ( ) | ( ) | ( ) | ( ) | ( ) |
| 1. Wenn ich einmal angefangen habe, etwas zu tun, höre ich nicht damit auf, bis ich fertig bin. | ( ) | ( ) | ( ) | ( ) | ( ) |
| 1. Ich nutze die guten Tage mit weniger Schmerzen aus, um mehr Dinge zu erledigen. | ( ) | ( ) | ( ) | ( ) | ( ) |
| 1. Ich erledige Dinge langsamer und kann so Energie sparen, die ich für andere Dinge nutzen kann, die mir etwas bedeuten. | ( ) | ( ) | ( ) | ( ) | ( ) |
| 1. Ich mache für gewöhnlich zahlreiche Ruhepausen, um weniger Schmerzen zu haben. | ( ) | ( ) | ( ) | ( ) | ( ) |
