# Supplementary material for: Cross-cultural adaptation and psychometric evaluation of a German version of the Activity Patterns Scale (APS-GE) in a large sample of patients with chronic musculoskeletal pain
Source: Front Pain Res (Lausanne). 2025 Jun 13;6:1570432. doi: 10.3389/fpain.2025.1570432 (PMC12202367; doi:10.3389/fpain.2025.1570432)
Supplement: Supplementary file 2 [file Table3.docx]

| **Supplementary Table 3.** Analysis of the relative contributions of different variable sets (sociodemographic and clinical variables, catastrophizing, pain-related activity patterns assessed by established questionnaires and by the APS-GE) to the explanation of variance in the disability composite score by hierarchical multiple regression analysis. | | | | |
| --- | --- | --- | --- | --- |
| **Variables entered upon each step (N= 534)^a^** | **Adjusted R^2^** | **R^2^_change_ (p-value)** | **β in final model (only statistically significant predictor variables shown)** | **p-value of final β** |
|  |  |  |  |  |
| **Step 1**: Highest school-leaving qualification | .026 | - | - .166 (highest school-leaving qualification) | p< .001 |
|  |  |  |  |  |
| **Step 2**: Widespread pain index (WPI), somatic symptom severity (SSS), outpatient psychotherapy (no. of sessions) | .251 | .229 (p< .001) | - .159 (highest school-leaving qualification)  .203 (widespread pain index)  .346 (somatic symptom severity) | p< .001 p< .001  p< .001 |
|  |  |  |  |  |
| **Step 3**: Catastrophizing (PCS) | .402 | .151 (p< .001) | - .087 (highest school-leaving qualification)  .183 (widespread pain index)  .185 (somatic symptom severity)  .430 (catastrophizing) | p= .011  p< .001 p< .001  p< .001 |
|  |  |  |  |  |
| **Step 4**: Activity avoidance (FABQ) | .413 | 0.012 (p= .001) | - .072 (highest school-leaving qualification)  .177 (widespread pain index)  .191 (somatic symptom severity)  .385 (catastrophizing)  .120 (activity avoidance, FABQ) | p= .035  p< .001  p< .001 p< .001  p= .001 |
|  |  |  |  |  |
| **Step 5**: Avoidance of physical activity, avoidance of social activities, endurance (AEQ) | .429 | .019 (p< .001) | - .078 (highest school-leaving qualification)  .179 (widespread pain index)  .166 (somatic symptom severity)  .356 (catastrophizing)  .083 (activity avoidance, FABQ)  .115 (avoidance of social activities, AEQ) | p= .021  p< .001  p< .001 p< .001  p= .029  p= .007 |
|  |  |  |  |  |
| **Step 6**: Pain avoidance, activity avoidance, task-contingent persistence, excessive persistence, pain-contingent persistence, pacing- increasing activity, pacing- conserve energy, pacing- pain reduction (APS-GE) | .525 | .102  (p< .001) | - .093 (highest school-leaving qualification)  .143 (widespread pain index)  .122 (somatic symptom severity)  .297 (catastrophizing)  .100 (avoidance of social activities, AEQ) .356 (activity avoidance, APS-GE) | p= .003  p< .001  p< .001 p< .001  p= .013  p< .001 |
|  |  |  |  |  |
| **Adjusted R^2^** **for final model** | 0.538 | - | - .097 (highest school-leaving qualification)  .137 (widespread pain index)  .166 (somatic symptom severity)  .300 (catastrophizing)  .070 (avoidance of social activities, AEQ)  .350 (activity avoidance, APS-GE) | p= .001  p< .001  p< .001 p< .001  p= .030  p< .001 |
| AEQ, Avoidance-Endurance Questionnaire; FABQ, Fear-Avoidance Beliefs Questionnaire; PCS, Pain Catastrophizing Scale; ^a^Hierarchical multiple linear regression analysis was performed on complete data sets only; predictor variables individually significantly associated with the disability composite score (mean of z-stand. PDI sumscore and z-stand. CPGQ disability sumscore) were entered in six steps; R^2^_change_= Increase in explained variance by step; adjusted R^2^, = R^2^-(k-1)/ (n-k)*(1- R^2^) where n=no. observations, k=no. independent variables. Level of significance was set to p=0.05. | | | | |

**Cross-cultural adaptation and** **psychometric evaluation of a German version of the Activity Pattern Scale (APS-GE) in a large sample of patients with chronic musculoskeletal pain**
