# Supplementary material for: Cross-cultural adaptation and psychometric evaluation of a German version of the Activity Patterns Scale (APS-GE) in a large sample of patients with chronic musculoskeletal pain
Source: Front Pain Res (Lausanne). 2025 Jun 13;6:1570432. doi: 10.3389/fpain.2025.1570432 (PMC12202367; doi:10.3389/fpain.2025.1570432)
Supplement: Supplementary file 3 [file Table4.docx]

| **Supplementary Table 4.** Analysis of the relative contributions of different variable sets (sociodemographic and clinical variables, catastrophizing, pain-related activity patterns assessed by established questionnaires and by the APS-GE) to the explanation of variance in the psychological distress composite by hierarchical multiple regression analysis. | | | | |
| --- | --- | --- | --- | --- |
| **Variables entered upon each step (N= 534)^a^** | **Adjusted R^2^** | **R^2^_change_ (p-value)** | **β in final model (only statistically significant predictor variables shown)** | **p-value of final β** |
|  |  |  |  |  |
| **Step 1**: Highest school-leaving qualification | .015 | - | - .131 (highest school-leaving qualification) | p= .002 |
|  |  |  |  |  |
| **Step 2**: Widespread pain index (WPI), somatic symptom severity (SSS), outpatient psychotherapy (no. of sessions) | .407 | .394 (p< .001) | - .126 (highest school-leaving qualification)  .610 (somatic symptom severity) | p< .001  p< .001 |
|  |  |  |  |  |
| **Step 3**: Catastrophizing (PCS) | .576 | .169 (p< .001) | .439 (somatic symptom severity)  .455 (catastrophizing) | p< .001  p< .001 |
|  |  |  |  |  |
| **Step 4**: Activity avoidance (FABQ) | .575 | 0.000 (p= .709) | .439 (somatic symptom severity)  .450 (catastrophizing) | p< .001 p< .001 |
|  |  |  |  |  |
| **Step 5**: Avoidance of physical activity, avoidance of social activities, endurance (AEQ) | .588 | .014 (p< .001) | .417 (somatic symptom severity)  .427 (catastrophizing)  - .117 (avoidance of physical activities, AEQ)  .123 (avoidance of social activities, AEQ) | p< .001  p< .001  p= .002 p< .001 |
|  |  |  |  |  |
| **Step 6**: Pain avoidance, activity avoidance, task-contingent persistence, excessive persistence, pain-contingent persistence, pacing- increasing activity, pacing- conserve energy, pacing- pain reduction (APS-GE) | .594 | .012  (p= .045) | .392 (somatic symptom severity)  .413 (catastrophizing)  - .097 (avoidance of physical activities, AEQ)  .105 (avoidance of social activities, AEQ)  .094 (excessive persistence)  - .103 (pacing- conserve energy) | p< .001  p< .001  p= .029 p= .005  p= .007  p= .047 |
|  |  |  |  |  |
| **Adjusted R^2^** **for final model** | 0.598 | - | .396 (somatic symptom severity)  .437 (catastrophizing)  - .099 (avoidance of physical activities, AEQ)  .103 (avoidance of social activities, AEQ)  .094 (excessive persistence) | p< .001  p< .001  p= .006 p= .004  p= .002 |
| AEQ, Avoidance-Endurance Questionnaire; FABQ, Fear-Avoidance Beliefs Questionnaire; PCS, Pain Catastrophizing Scale; ^a^Hierarchical multiple linear regression analysis was performed on complete data sets only; same set of predictor variables for psychological composite score (mean of z-stand. DASS depression, DASS anxiety, DASS stress and PANAS negative affect) as for disability composite score; R^2^_change_= Increase in explained variance by step; adjusted R^2^, = R^2^-(k-1)/ (n-k)*(1- R^2^) where n=no. observations, k=no. independent variables. Level of significance was set to p=0.05. | | | | |

**Cross-cultural adaptation and** **psychometric evaluation of a German version of the Activity Pattern Scale (APS-GE) in a large sample of patients with chronic musculoskeletal pain**

**Anne Kästner^1^, Margarete Donhauser^1^****, Inga von Freytag-Löringhoff^1^, Frank Petzke^1^**

**^1^Department of Anesthesiology, Pain Clinic, University Hospital, Georg-August-University of Göttingen, Germany**
